# The human parvovirus B19/human immunodeficiency virus co-infection in healthy eligible voluntary blood donors at the Blood Transfusion National Center in Kinshasa

**DOI:** 10.11604/pamj.2020.35.69.21018

**Published:** 2020-03-10

**Authors:** Chabo Byaene Alain, Lufimbo Katawandja Antoine, Bizeti Nsangu Bizette, Pambu Dahlia, Tshibuela Beya Dophie, Muwonga Masidi Jérémie, Kayembe Nzongola-Nkasu Donatien, Ahuka Mundeke Steve

**Affiliations:** 1 Department of Immunology and Regenerative Medicine, Faculty of Pharmacy, Mansoura University, Mansoura, Egypt; 2 Department of Medical Biology, Faculty of Medicine, University of Kinshasa, Kinshasa, Democratic Republic of Congo

**Keywords:** Parvovirus B19, co-infection, blood donors, HIV, Kinshasa

## Abstract

**Introduction:**

Parvovirus B19 (PVB19) is one of several viruses transmissible by blood transfusion. Levels of exposure to PVB19 among HIV-infected voluntary blood donors are comparable to those among HIV-negative controls because, in blood donors, the PVB19 infection is transmitted mainly via the respiratory route. Thus, we hypothesize that the seroprevalence of PVB19 in HIV-positive blood donors is equal to the seroprevalence of PVB19 in HIV-negative blood donors. The objective of this study was to compare the seroprevalence of PVB19 between asymptomatic HIV-positive and HIV-negative blood donors.

**Methods:**

A random sample of 360 eligible blood donors were firstly examined for HIV antibodies by using ELISA automaton and so were categorized as HIV-positive donors and HIV-negative donors. Then the two categories of donors were examined for PVB19 IgG and IgM by using ELISA kits. The seroprevalence of PVB19 in HIV-positive donors was compared to that of HIV-negative donors by using chi-square test or Fisher's exact test. All statistical analyzes were performed with SPSS 21.

**Results:**

The prevalences of PVB19 IgG and IgM in HIV-positive blood donors were 92.1% (35 of 38) and 44.7% (17 of 38), respectively and those in control group were 89.1% (287 of 322) and 46.3% (149 of 322), respectively. But for both IgG and IgM the difference was not statistically significant (p > 0.05).

**Conclusion:**

This research confirms our hypothesis: the seroprevalence of PVB19 in HIV-positive blood donors is equal to the seroprevalence of PVB19 in HIV-negative blood donors.

## Introduction

There are several viruses responsible for infections transmitted by blood transfusion including hepatitis B virus, hepatitis C virus, human T Lymphotropic Virus (HTLV 1 and 2), Cytomegalovirus (CMV), human immunodeficiency (HIV 1 and 2) and Parvovirus B19 (PVB19) [1-8]. The transmission of PVB19 is mainly via the respiratory route, from the mother to the fetus, through the blood product and through transplants [9]. Iatrogenic transmission occurs through blood transfusion or organ transplantation from the seropositive donor. Iatrogenic transmission is favored by three important features of the virus: (i) persistent virus infection in the bone marrow of an asymptomatic carrier [10]; (ii) prolonged replication after infection or initial reinfection [11]. In immunocompromised patients, infection may persist by reactivation or reinfection [12]; (iii) the resistance of the virus to many inactivation methods used in the manufacture of blood derivatives, plasma derivatives and labile blood products [13-17]. The transmission of PVB19 by blood transfusion occurs during the period of high viraemia in the donor. Viremia occurs approximately 1 week after primary infection and persists at elevated titres of up to 10^14^ viral particles/mL in plasma for approximately 7 days [13,18]. Several cases of transfusion transmission of PVB19 have been reported, and many contaminated blood donations have been retrospectively or prospectively detected [15, 16, 19-22]. PVB19, also called erythrovirus B19, is the basis of several syndromes whose clinical manifestations may be moderate or severe. They vary according to the hematological and immunological status of the infected person [23].

The child, the pregnant woman, the persons suffering from chronic hemolysis and the immunocompromised, are the most affected persons [24]. In the immunocompetent, the infection is usually asymptomatic or nonspecific. It can cause subclinical and limited aplasia of red blood cells followed by skin rash or arthralgia. The best known clinical manifestation in children is erythema infectiosum (fifth pediatric eruptive disease) [25]. It is a moderately intense facial erythema with cheeks, the prodrome of which is characterized by fever, colds, headache and nausea. An association between PVB19 infection and arthropathy was established in 1985. In non-immunized pregnant women, PVB19 carries a risk of fetal anasarca. In people with chronic hemolysis, such as sickle cell and thalassemic and not yet immunized, PVB19 can cause profound central anemia. In immunocompromised patients, such as patients receiving chemotherapy or people infected by HIV, PVB19 infection may be the cause of chronic anemia (red blood cell aplasia) following continuous and uncontrolled replication of the virus causing destruction of erythroblasts [26]. About 5% of adults and 10% of children suffering with hematological malignancy and chemotherapy are chronically infected with PVB19 and therefore develop severe and sometimes fatal cytopenia [27]. The risk of PVB19 transmission in HIV-infected people is comparable to the risk in HIV-negative controls, since PVB19 infection is transmitted through the respiratory route [28]. Thus, we hypothesize that the seroprevalence of PVB19 in HIV-positive blood donors is equal to the seroprevalence of PVB19 in HIV-negative blood donors. The objective of this study is to compare the seroprevalence of PVB19 between HIV-positive blood donors and HIV-negative blood donors.

## Methods

**Framework, type and duration of the study:** this is a cross-sectional descriptive study, which took place in the period between 2016-2017, at the Blood Transfusion National Center (BTNC) in Kinshasa.

**Study population:** the source population consisted of donors eligible for blood donation who were selected during the mobile collection campaigns organized by the BTNC. Donor selection was based on the criteria below.

**Inclusion criteria:** to be included in the study, one must first be eligible for blood donation according to the World Health Organization (WHO) recommendations and then give informed consent. The eligibility criteria are to be adult under 65 years old, healthy and have responsible sexual behavior.

**Exclusion criteria:** any blood donor who did not give informed consent or who did not meet the inclusion criteria above was excluded from the study. Thus, blood donors with chronic illness such as diabetes or high blood pressure, alcoholic or addicted to tobacco and menstruating women were excluded from this study.

**Sampling technique:** our sampling was probabilistic. The minimum sample size was calculated by the following formula [29]:

$$n=\frac{z^{2}p(1-p)}{e^{2}}$$

n is the sample size; z is a constant from the normal distribution at a certain confidence level (usually 95% and z = 1.96); p is the assumed prevalence of the investigated disease; e is the margin of sampling error chosen or the degree of precision desired. As the prevalence of PVB19 infection in blood donors in the DRC is unknown, the prevalence of 94% in blood donors, reported in a previous study in Nigeria [30] served as a baseline. Indeed, according to this study, 83 out of 88 blood donors had anti-parvovirus B19 antibody: 13 donors were IgM positive; 33 IgG positive and 37 positive for both subtypes (IgM and IgG). Thus, the size of our sample for a degree of accuracy of 5% is:

$$\text{n}\geq \frac{{\left(1.96\right)}^{2}}{{\left(0.05\right)}^{2}}\times 0.94(1-0.94)i.e.\text{n}\geq \text{87}$$

The minimum number required is 87. Taking into account non-respondents, the sample size was increased by 10%. Thus, the final size was 96. We selected 360 donor samples;

**Collection of data:** sociodemographic data included age, sex, province of origin, district and profession/occupation; biological data from blood donors included PVB19 IgM and IgG serological status. All these data were collected on a collection sheet pre-established by the National Program of Blood Transfusion (NPBT) in the DRC;

**Sampling, conservation and transport of samples:** the samples were taken after collection of blood in the bag. Once the pouch was filled, the tubing was clamped with two clips and then cut between the clamps, flush with the needle. Then, about 10 mL of blood was taken from tubes without anticoagulant. During the mobile campaigns, the samples obtained were transported to the cold storage tanks, on the same day, at the Immuno-Hematology Laboratory of NBTC. Once in the laboratory, the samples were first centrifuged using the Thermo CL10 centrifuge at 3000 rpm for 5 minutes. The search for four biological markers of qualification (HIV, hepatitis B, hepatitis C and syphilis) was carried out directly and then the rest of the serum was aliquoted and then kept at -80°C in a Dometic UF 755 G refrigerator. Aliquots kept in the Immuno-hematology laboratory of the NCBT were transported to the laboratory of virology, also known as the Cremer laboratory, of the University Clinics of Kinshasa for the detection of anti-PVB19 antibodies.

**Biological analyzes:** we used an RIDA^®^ RF-Absorbens Kits (Z0202), RIDASCREEN^®^ Parvovirus B19 IgG Kits (K60^21^) and RIDASCREEN^®^ Parvovirus B19 IgM Kits (K6031). Serological analyzes were carried out at the Laboratory of Virology (Cremer laboratory) of the Faculty of Medicine of the University of Kinshasa. PVB19 infection was investigated in all subjects included in this study by the detection of PVB19 IgM and IgG antibodies using the enzyme-linked immunosorbent assay (ELISA) with parvovirus B19 RIDASCREEN kit, with sensitivity of 100% for IgG and 84.4% for IgM, and with specificity of 100% for IgG and 95.5% for IgM, according to information obtained from the manufacturer of the reagent. All handling was carried out in accordance with the manufacturer's recommendations. Before interpreting the results, we calculated the average optical densities (OD) of the controls and ensured that it was within the range of values given on the manufacturer's data sheet. If the difference between two individual measurements was more than 20% compared to the mean OD of the controls, the test was to be resumed. The negative control should indicate an OD less than 0.3. Before the analysis, the test value of the blank reagent was deduced from all measured values. The OD thus obtained was located in the different rows (lower, doubtful, positive) of the column corresponding to the mean OD of the controls. The test was negative, doubtful or positive when this OD was in the corresponding range on the manufacturer's data sheet.

**Results interpretation:** results for PVB19 IgG and IgM were reported as either 'positive' or 'negative'. Results were interpreted as follows: positive IgG and negative IgM indicate past infection; positive IgG and IgM indicate infection within the last 7-120 days; negative IgG and positive IgM indicate acute/recent infection; negative IgG and IgM indicate that the donor is not immune and that no evidence of acute/recent infection is identified.

**Statistical analyzes:** the information collected was coded and stored in a database using Microsoft Excel version 2007. The database contains sample identification numbers, donor socio-demographic information and results of various serological tests. Data processing and analysis were performed using SPPS software version Windows 21.0 The chi-square test or Fisher's exact test was used to compare the seroprevalence of PVB19 between HIV-positive donors and HIV-negative donors. Student's t test was used to compare average ages between HIV-positive donors and HIV-negative donors. The odds ratio (OR) and its 95% confidence interval (95% CI) were used to evaluate the association between PVB19 infection and HIV infection. The value of p <0.05 was considered statistically significant.

**Ethical consideration:** informed consent was sought and obtained from each donor before inclusion. The subjects included in this study were not exposed to particular risks related to manipulation (sampling). This study received the approval of the ethics committee of the School of Public Health of the University of Kinshasa which is in accordance with the Code of Ethics of World Medical Association (Declaration of Helsinki involving use and handling of human subjects). The number of the letter of approval is ESP/CE/018/16.

## Results

Table 1 shows the general characteristics of the study population. Among the 360 blood donors included in this study, 38 (10.6%, 95% CI, 7.4% - 13.8%) were HIV-positive and 3^22^ (89.4%, 95% CI, 86.2% - 92.6%) were HIV-negative. The 18 to 31 age group was the most represented at 50% in HIV-positive blood donors and 51.2% in HIV-negative blood donors. Male donors were the most numerous with a gender ratio of 4.4 for HIV-positive and 5.2 for HIV-negative. The majority of donors were single and accounted for 68.4% of HIV-positive donors and 64% of HIV-negative donors. Tshangu district hosts the largest percentage of donors: 31.6% of HIV-positive donors and 34.5% of HIV-negative donors live in Tshangu district. The majority of donors do liberal work and represent 50% of HIV-positive donors and 45.6% of HIV-negative donors. In order to determine the seroprevalence of PVB19 antibody, PVB19 specific antibodies (IgG and IgM) were investigated in serum samples from 38 HIV-positive blood donors and 322 HIV-negative blood donors. As shown in Table 2, among the 38 HIV-positive samples tested for PVB19 specific immunoglobulins, nineteen (50%, 95% CI, 34% - 66%) were positive for IgG; one (2.6%, 95% CI, -2.4% - 7.6%) was positive for IgM; sixteen (42.1%, 95% CI, 26.4% - 57.8%) were positive for both antibodies and two (5.3%, 95% CI, -1.8% - 12.4%) were negative for both subtypes. Among the 322 HIV-negative samples tested for PVB19 specific immunoglobulins, 156 (48.4%, 95% CI, 42.9% - 53.9%) were positive for IgG; 18 (5.6%, 95% CI, 3.1% - 8.1%) were positive for IgM; 131 (40.7%, 95% CI, 35.3% - 46.1%) were positive for both antibodies and 17 (5.3%, 95% CI, 2.9% - 7.7%) were negative for both subtypes. The prevalences of PVB19 IgG and IgM in HIV-positive blood donors were 92.1% (35 of 38, 95% CI, 83.5% - 100.7%) and 44.7% (17 of 38, 95% CI, 28.9% - 60.5%), respectively and those in the control group were 89.1% (287 of 322, 95% CI, 85.7% - 92.5%) and 46.3% (149 of 322, 95% CI, 40.9% - 51.7%), respectively.

**Table 1:** General characteristics of the study population

Variable	HIV+	HIV-	Total	Observation
**Age (years)**				
18-31	19	165	184	χ^2^ = 2.43, p>0.05
32-45	11	117	128	
>45	8	40	48	
Total	38	322	360	
**Profession**				
Liberal	19	147	166	χ^2^ = 2.825, p>0.05
Without occupation	8	108	116	
Others*	11	67	78	
Total	38	322	360	
**Address (District)**				
Tshangu	12	111	123	χ^2^ = 2.408, p>0.05
Funa	10	91	101	
Lukunga	7	74	81	
Mont Amba	9	46	55	
Total	38	322	360	
**Sex**				
Male	31	270	301	χ^2^ = 0.128, p>0.05
Female	7	52	59	
Total	38	322	360	
**Civil status**				
Single	26	206	232	χ^2^ = 0.293, p>0.05
Married	12	116	128	
Total	38	322	360	

* Others: students and employees

**Table 2:** PVB19 Seroprevalence and HIV

PVB19	HIV+	HIV-	Total	Observation
**IgG**				
IgG+	35	287	322	χ^2^ = 0.3056, p>0.05
IgG-	3	35	38	Fisher test: p=0.57653
Total	38	322	360	OR=0.7543
				95% CI [0.2661-2.6451]
**IgM**				
IgM+	17	149	166	χ^2^ = 0.033, p>0.05
IgM-	21	173	194	Fisher test: p=1
Total	38	322	360	OR=0.9401
				95% CI [0.4476-1.9493]
**IgG/IgM**				
IgG+/IgM-	19	156	175	χ^2^ = 0.033, p>0.05
IgM+/IgG-	1	18	19	Fisher test: p=0.865652
				OR=1.0639
				95% CI [0.5119-2.2112]
IgG+/IgM+	16	131	147	χ^2^ = 0.594, p>0.05
				Fisher test: p=0.7062
				OR=0.4572
				95% CI [0.0107-3.068]
IgG-/IgM-	2	17	19	χ2 = 0.028, p>0.05
				Fisher test: p=0.86332
Total	38	322	360	OR=1.0602
				95% CI [0.4999-2.2051]
				χ^2^ = 0, p>0.05
				Fisher test: p=1
				OR=0.9967
				95% CI [0.1074-4.4824]

## Discussion

We used the HIV ELISA test allowed us to classify blood donors into two categories: HIV-positive donors and HIV-negative donors. Table 1 shows the socio-demographic characteristics of the two groups of donors. The chi-square test of homogeneity shows that the two categories of donors, i.e. HIV-positive donors and HIV-negative donors come from the same population. Sociodemographic characteristics were not different in the group of HIV-positive donors compared to the control group.

$$(x_{c}^{2}<3.84;\text{p>0.05)}$$

Then we looked for anti-PVB19 antibodies in both blood donor categories. Table 2 shows the frequency of anti-PVB19 antibodies in both donor groups. The chi-square test of independence shows that PVB19 infection and HIV infection are independent.

$$(x_{c}^{2}<3.84;\text{p>0.05)}$$

Among the 360 blood donors, 36 had anti-PVB19 antibodies and anti-HIV antibodies, so the frequency of coinfection is 10%. Surprisingly, these 36 donors were considered eligible for blood donation. PVB19 has been described as a cause of chronic anemia and a variety of other diseases in immunosuppressed patients, including those infected with the human immunodeficiency virus (HIV) [31-35]. Christensen *et al.* implicated parvovirus infection as the cause of cystitis in a patient with HIV infection [36,37]. Thus, clinical manifestations of all these diseases may not have been carefully investigated during the donors’ clinical selection. However, the literature confirms that in HIV-positive patients without AIDS, by contrast, PVB19 infection evolves as an exanthematous disease, or may be entirely asymptomatic [38]. If therefore PVB19-HIV co-infection was asymptomatic for the 36 blood donors, it is quite normal that they have been found eligible for blood donation during the clinical screening of donors. Among the 38 HIV-positive blood donors, 36 had anti-PVB19 antibodies (IgG and IgM), meaning a seroprevalence of 94.7%. In 1997, Raguin *et al.* determined the prevalence of PVB19 in 55 HIV-infected patients. Anti-PVB19 IgG antibodies were detected in 53/55 (96%) of the serum samples and anti-PVB19 IgM antibodies were detected in only five (10%) patients who were also positive for anti-PVB19 IgG antibodies [39]. Our results agree with those of Raguin.

The seroprevalence of anti-PVB19 IgG was 92.1% in HIV-infected donors, which was not significantly higher than that (89.1%) in the normal blood donor controls (p >0.05). These results are in agreement with the findings by Van Elsacker *et al.* [40] and contrast those published by Bremner *et al.* [41], Naides *et al.* [42], Zuckerman *et al.* [43] and [35] Miao *et al.* In fact, the study conducted in 1996 by Van Elsacker *et al.* also indicated that there was no statistically significant difference in seroprevalence of PVB19-specific IgG between HIV-infected patients and male blood donors. Studies by Bremner, Naides, Zuckerman *et al.* involving small numbers of patients, indicated a higher prevalence of IgG antibodies to PVB19 among HIV-infected individuals. Concerning Miao *et al.* study, the seroprevalence of IgG against PVB19 in HIV-infected individuals was significantly lower than that in the normal blood donor controls because patients infected with HIV may be incapable of producing IgG antibodies against PVB19 [44]. It seems that the seroprevalence of IgG is inversely correlated with the degree of immunodeficiency [35]. Patients with a CD4 count of over 300x10^6^ cells/L are usually capable of producing neutralizing antibodies [45]. In the present paper we studied asymptomatic blood donors.

Although we could not determine the CD4 T-cell count in order to elucidate the subjects' immune status, the HIV infection in these individuals were described as clinical stage 1 (asymptomatic), according to the revised WHO clinical staging of HIV/AIDS for adults and adolescents [46]. As the current study group was asymptomatic and recently diagnosed with HIV infection, this seems likely explanation to the equality of anti-PVB19 IgG seroprevalence between HIV-infected donors and normal blood donors. Another potential explanation of this PVB19 seroprevalence equality between the HIV-positive and HIV-negative blood donors is that HIV-infected blood donors may have been exposed to PVB19 and therefore developed anti-PVB19 IgG antibodies before their HIV infection. In fact the results of Dockrell *et al.* study suggest that, although HIV-positive individuals with decreased CD4 counts may have less effective immune response to T-lymphocyte-dependent antigens, they may still retain antibodies to antigens to which they have previously been exposed [47]. This explanation is likely because, as shown in Table 2, two out of 38 HIV+ donors have never been exposed to PVB19 and are therefore IgG-/IgM-; while 35 out of 38 HIV+ donors are IgG+. PVB19 has a worldwide distribution. The literature shows that the infection occurs normally in childhood [48]. In adulthood, donors already have IgG antibodies against PVB19. The degree of immunodeficiency and other confounding factors could explain the difference found in the IgG seroprevalence in different studies [45]. These variations in seroprevalence might be explained by seasonal, epidemiological or demographical characteristics that resulted in different rates of exposure to the virus [49].

Confounding factors may be the number of individuals included in the study, the sampling season. Epidemiological studies have shown that PVB19 activity occurs periodically, commonly in the form of outbreaks in the late spring and summer. These outbreaks represent an occasion in which susceptible individuals are at a higher risk of contracting PVB19 infection [50]. This study has limitations. The sensitivity and specificity of the ELISA kit were less than 100% for the PVB19 IgM antibody. This leads to false positives and false negatives for this antibody. However, this does not result in a difference in seropositivity between the two groups of donors. Another limitation may be the low representativeness of HIV positive donors. Despite these limitations, this was the first time that human PVB19 in HIV+ and HIV- blood donors in Kinshasa, Democratic Republic of Congo had been investigated. The control population (HIV-) was identical to the subjects (HIV+), there was no difference in age, no difference in gender distribution and other demographical characteristics that resulted in different rates of exposure to the virus.

## Conclusion

Our results indicate that PVB19 seroprevalence in HIV-seropositive blood donors is not significantly different from PVB19 seroprevalence in HIV-seronegative blood donors and thus confirm our hypothesis.

### What is known about this topic

- There is no data on HIV-PVB19 coinfection in the Democratic Republic of Congo.

### What this study adds

- This article provides data on HIV-PVB19 coinfection in the Democratic Republic of Congo and shows that the seroprevalence of PVB19 in HIV-seropositive blood donors is equal to the seroprevalence of PVB19 in HIV-seronegative blood donors.

## Competing interests

The authors declare no competing interests.
